# Training Load, Maturity Timing and Future National Team Selection in National Youth Basketball Players

**DOI:** 10.3390/jfmk7010021

**Published:** 2022-02-11

**Authors:** Jorge Arede, Tomás T. Freitas, David Johnson, John F. T. Fernandes, Sean Williams, Jason Moran, Nuno Leite

**Affiliations:** 1 Department of Sports Sciences, Exercise and Health, University of Trás-os-Montes and Alto Douro, 5001-801 Vila Real, Portugal; nleite@utad.pt; 2 School of Education, Polytechnic Institute of Viseu, 3504-501 Viseu, Portugal; 3 Department of Sports, Higher Institute of Educational Sciences of the Douro, 4560-708 Penafiel, Portugal; 4 School of Sports Sciences, Universidad Europea de Madrid, Campus de Villaviciosa de Odón, 28670 Madrid, Spain; 5 UCAM Research Center for High Performance Sport, Catholic University of Murcia (UCAM), 30107 Murcia, Spain; tfreitas@ucam.edu; 6 NAR—Nucleus of High Performance in Sport, São Paulo 04753-060, Brazil; 7 Faculty of Sport Sciences, Catholic University of Murcia (UCAM), 30107 Murcia, Spain; 8 Department for Health, University of Bath, Bath BA2 7AY, UK; dmj32@bath.ac.uk (D.J.); sw365@bath.ac.uk (S.W.); 9 School of Sport and Health Sciences, Cardiff Metropolitan University, Cardiff CF23 6XD, UK; jfmtfernandes@hotmail.co.uk; 10 School of Sport, Rehabilitation and Exercise Sciences, University of Essex, Colchester CO4 3SQ, UK; jmorana@essex.ac.uk; 11 Research Center in Sports Sciences, Health Sciences and Human Development, CIDESD, University of Trás-os-Montes and Alto Douro, 5001-801 Vila Real, Portugal

**Keywords:** talent, puberty, growth, maturation, adolescence, rate of perceived exertion

## Abstract

Despite its importance to the management of training stress, monotony and recovery from exercise, training load has not been quantified during periods of intensity training in youths. This study aimed to (1) examine and quantify the training load (TL) in youth national team basketball players during a 2-week training camp according to maturity timing and (2) determine which parameters were related to under-18 (U18) national team selection. Twenty-nine U-16 national team basketball players underwent an anthropometric assessment to determine maturity timing. Players were categorised by maturity timing (early vs. average), whilst TL parameters during a 2-week training camp (i.e., 21 sessions) prior to FIBA U16 European Championship were used for group comparison and to predict future U-18 national team selection. The early-maturing players, who were taller and heavier (*p* < 0.05), experienced greater training strain in week 1 (*p* < 0.05) only. Irrespective of maturity timing, training loads in week 2 were predictive of onward selection for the U-18 national team. Conclusion: Based on present findings, practitioners are encouraged to develop their athletes’ ability to tolerate high weekly loads, but also to be mindful that athletes’ perceived exertion during national team training may be influenced by maturity timing.

## 1. Introduction

The national training camp comprises a very limited time period and a congested training and/or match schedule aiming to enhance player readiness, and increase the likelihood of winning fixtures [1]. The high physical demands and training load compared with club settings may result in higher injury rates during national team training camp [2]. Thus, to have all players available to practice and compete prior to international competition is important to align injury risk factors (e.g., accumulated fatigue, reduced recovery time, and training load) with potential mitigation strategies [3]. In this regard, the use of simple and reliable method to quantify training demands, such as rating of perceived exertion (RPE) can offer an appropriate load monitoring, underpinning the management of the likelihood of injury and the improvement of athlete’s performance [4].

Training load monitoring can be particularly important when most of youth basketball national team players can be classified in terms of injury risk stratification as “High risk athlete” or “Load sensitive” [5], when experience higher training loads during national team training camp [1], and most of national level players only practiced basketball since early age (i.e., 10 years old) [6]. This pattern of early engagement in youth sport includes few opportunities to experience a variety of load adaptive stimulus, resulting in fully develop neuromuscular patterns which protect against injury [5]. In this regard, national level players who specialized early engagement in youth sport (6 to 10 years old) shown worse jumping and sprinting abilities comparing with their peers [6]. Moreover, the engagement in early sports specialisation before pubertal growth may deteriorate biomechanical qualities that can propagate through maturational development in young athletes [5].

A key contributor to the physical and athletic development of youth is the process of biological maturation [7]. Maturation refers to the progression towards a mature state and can be defined in terms of status, timing, and tempo [8]. Maturity timing relates to when a particular maturational event occurs (e.g., the timing of peak height velocity [PHV]) and can be used to classify individuals as ‘early’, ‘on-time’ or ‘late’ for the purposes of training and talent development. Early maturing youths tend to be taller and heavier and possess greater absolute and relative lean mass than their less mature peers, and perform better on tests of strength, speed, and power [7,9,10]. As such, earlier maturation has been associated with a selection bias in a team-sports whereby early maturing individuals are preferentially chosen over these less mature peers [11,12,13,14]. Additionally, in basketball, there are pronounced maturity-associated differences in anthropometric, physical [14] and game performance-related variables [11,15] which can result in coaches favouring and preferentially selecting early maturing players [11]. However, these advantages are transient [16] with maturity-associated differences in both size and function having shown to be attenuated, and in some cases reversed, in early adulthood [17]. Moreover, early maturing and taller basketball players show a decreased ability to use lower limb muscles, illustrated by their reduced performance in high-intensity actions (i.e., jumping, sprinting, change of direction, etc.), but also a significant bilateral asymmetry (>15%), reduced aerobic fitness [18], and poorer functional performance [19], which can modulate their ability to adapt to the training stimulus [18]. Therefore, the frequent monitoring (i.e., daily to weekly) [5], including quantifying training load can support the adequate prescription of training load based on their tolerance, in order to enhance resilience to the demands of high-level training and competition, as occurs in national team setting [5]. Despite the training load monitoring based on RPE-derived variables has been particularly used considering maturity status (i.e., pubertal period) [20], recently this strategy has been suggested in post-pubertal period in order to mitigate the risk of injury [21], reinforcing its usefulness in “Load Sensitive” players, such as youth basketball national team players.

Talent selection strategies in youth basketball national teams is a complex, multifactorial and dynamic process which varies according national sporting organisations and federations [22]. Different variables have been suggested as determinant to select and retain players within national team selection process, such as initial selection age, relative age effect [23], game performance [19], training experience and biological maturation [14]. Furthermore, a previous study demonstrated a positive influence of the interaction of physical, game, and training (e.g., muscle soreness)-derived variables be a starter during official games of youth basketball national team [1]. Despite this scant evidence, more studies are warranted to understand the role of training load variables in national team selection process. This is particularly important when talent development systems may impose a “survival of the fittest” mentality on their participants whereby the so-called “survivors” in a given sport (i.e., those who are selected) are those players who can cope with the highest physical demands and loads of intensive training, at the expense of those who may become injured as a result of these practices [24]. Consequently, ‘gifted’, yet less durable, young athletes may be denied a career in elite sport if their progress is curtailed by injuries or health problems related to the training load of their physical preparation programme [25]. This study aims to (1) examine and quantify the TL in youth national team basketball players during a 2-week training camp according to maturity timing and (2) determine which parameters were related to onward selection to the under-18 (U18) national team.

## 2. Materials and Methods

### 2.1. Participants

Twenty-nine U16 male basketball players (age = 16.0 ± 0.4 years) were selected by the Portuguese national coaching staff to participate in the 5-week national team training camp across three separate seasons (July/August in 2016, 2017, and 2018 years). The national coaching staff was comprised of the same individuals throughout these seasons and twelve different players were selected in each season. Players who missed more than 3 days due to injury, technical reasons or other issues were excluded from further analysis, to avoid substantial influence in training load parameters. Most players were of Portuguese ancestry (*n* = 24), with the exception of the players hailing from African countries (*n* = 5). Thereby, twenty-nine athletes were included in present study (2016, *n* = 12; 2017, *n* = 9; 2018, *n* = 8). These national team training camps prepared the players for participation in the FIBA U16 European Championship Division B in separate seasons. Written informed consent was obtained from all participants and their parents prior to this investigation. The study was approved by the institutional research ethics committee and conformed to the recommendations of the Declaration of Helsinki.

### 2.2. Procedures

All data were obtained at three consecutive yearly national training camps and were collected by the same investigator to ensure testing accuracy and reliability. In each season, the national training camp included forty-five team practices of ~2 h duration. The first two weeks national team training camp comprised 21 in-court training sessions (mean duration = 117.3 ± 15.3 min.; range = 82–154 min.). Training sessions occurred under the same environmental conditions (session time and indoor basketball court).

### 2.3. Anthropometric Data

Body mass, height and sitting height were recorded for the estimation of somatic maturation during National Team training camp. Body mass was assessed using a body fat monitor (Tanita BF-522W, Canaxi, Tokyo, Japan) calibrated to the nearest 0.1 kg. Height and sitting height were assessed using a portable stadiometer with measurements taken to the nearest 0.1 cm (Tanita BF-522W, Canaxi, Tokyo, Japan). All data collection techniques followed the guidelines outlined by the International Society for the Advancement of Kinanthropometry (ISAK) [26].

### 2.4. Somatic Maturation

Maturity offset (MO) was predicted using a non-invasive method appropriate for the age range of the sample, considering anthropometric data (body mass, standing height, leg length and sitting height) and chronological age [27,28]. The APHV was calculated by subtracting MO from the chronological age. Predicted adult height (PAH) was computed from the sum of an individual’s height at the time of the measurements and distance left to grow in height according to APHV (early, average and late) and MO [29]. Based on average APHV (14.0 years of age in males), the subjects were grouped into two maturity timing categories: early (APHV at 13 years old or before), and average (APHV between 13 and 15 years old) maturing [29]. The data available show underestimated adult height at 16 years old using a non-invasive method [29] compared with real values at full adult age [19], but also that the Mirwald method significantly overestimated adult height compared with the Khamis and Roche method [19].

### 2.5. Training Load

Data were collected from players (*n* = 12 each year) during the first 2-weeks of national team training camp in Portugal. Before the first session of each national team training camp, one familiarization session was carried out including the instruction on how to use the Borg CR10 scale. Subjects were verbally asked to rate their perceived exertion based on Borg CR10 scale, thirty minutes following the completion of each training session (session ratings of perceived exertion (sRPE)) [30]. Main researcher recorded the ratings of perceived exertion using a pen-and-paper data collection sheet. The training load (TL) was calculated by multiplying session duration (mins) by sRPE. Weekly TL was calculated using the sum of the TL for all training sessions performed in a given week [31]. Weekly training monotony (i.e., day to day training variability in a given week) was calculated from the average weekly TL divided by the standard deviation of the weekly TL [32]. Weekly training strain (i.e., overall stress of the training week) was calculated as the product of the weekly TL and weekly training monotony [32]. The absolute week-to-week change in load (W-TL) was calculated as the difference between TL in Week 2 and TL in Week 1 [33]. Moreover, the week-to-week load changes were also calculated by dividing the TL in Week 2 by the TL in Week 1 [34].

### 2.6. Statistical Analyses

Descriptive data are presented as mean ± SD. Normality of the data was confirmed using the Shapiro–Wilk test (*p* > 0.05). A Chi-Square test of independence was applied to identify associations between maturity timing in view of playing positions and year of national team training camp. Effect size (ES) resulting from Chi-Square test was estimated by calculating Cramer’s V (V) correlation coefficients, considering 0.10 as small effect, 0.30 as medium effect and 0.50 as large effect [35]. Independent samples *t*-tests were used to detect any between-groups differences. Effect sizes (ES) of the differences between maturity timing groups were evaluated using Hedges’ g correcting small sample biases [36]. Effect sizes were considered <0.2 trivial, >0.2–0.5 small, >0.5–0.8 medium, >0.8–1.3 large, and >1.3 very large [37]. The variables, for which significant differences were identified between groups, were used in a stepwise discriminant function analysis to determine what set of variables discriminated early and average maturing players. Finally, a stepwise discriminant analysis was conducted to identify a construct that best classified selected and non-selected players for further under-18 national team training camp (a year or two later). All analyses were performed using SPSS (SPSS, Inc., Version 24.0, Chicago, IL, USA).

## 3. Results

### 3.1. Player’s Demographics

The mean age, height and body mass of the included players was 16.04 ± 0.34 years (range: 15–16.50 years), 190.19 ± 7.06 cm (range: 174–204 cm) and 81.67 ± 10.78 kg (range: 63.90–106.20 kg), respectively. The mean age of age of peak height velocity and maturity offset was 13.26 ± 0.61 years (range: 11.80–14.30) and 2.67 ± 0.63 years (range: 1.50–4.00), respectively. No late-maturing athletes were identified. Analysis showed significant differences between maturity timing with respect to the playing position (χ^2^ (1, *n* = 29) = 2.24; *p* = 0.039; ϕCramer = 0.38), but not regarding further national team selection or year of national team training camp (all *p* > 0.05). Most of early-maturing players were inside players (i.e., players who use areas close to the basket to score), whereas most of average-maturing players were perimeter players (i.e., players who use the perimeter area to score). Regarding maturity timing distribution according to the years (χ^2^ (2, *n* = 29) = 3.63; *p* = 0.163), and further national team selection (χ^2^ (1, *n* = 29) = 1.72; *p* = 0.189) no differences were observed.

### 3.2. Training Load Description

**Figure 1:**
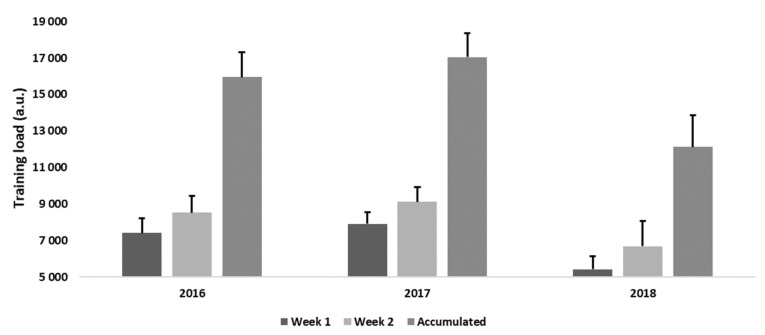
Training load patterns during 2-week national team training camp in different years.

There were significant differences in training load variables between the year and week (all *p* < 0.001) (Figure 1). Post hoc analysis revealed that in 2018 subjects experienced less accumulated training load than in 2016 and 2017 (all *p* < 0.001). Moreover, training load was higher in week 2 than 1, for all years (*p* < 0.001).

### 3.3. Between-Groups Differences

Results of the inferential analysis for anthropometric, and training load variables are displayed in Table 1. Early-maturing players were significantly taller, and heavier than average subjects (all *p* < 0.01) (Table 1). Moreover, early-maturing players had a significantly lower training experience, and experienced higher weekly training strain during Week 1 (all *p* < 0.05) (Table 1).

Discriminant analysis showed that sitting height (coefficient: 0.719), body mass (coefficient: 0.574), height (coefficient: 0.437), training experience (coefficient: −0.155) and training strain during Week 1 (coefficient: 0.090) discriminated between early- and average-maturing players (Table 2).

### 3.4. Further Selection

Finally, the construct obtained in the stepwise discriminant analysis provided a model that considered training load in Week 2 as the best discriminating variable between selected (*n* = 20) and non-selected (*n* = 9) players for U18 National Team (Table 3).

## 4. Discussion

The aims of the present study were to: (1) examine and quantify the TL in youth national team basketball players during a 2-week training camp according to maturity timing and (2) determine which parameters were related to onward selection to the under-18 (U18) national team. The main findings indicated that early maturing players were taller and heavier than their average-maturing counterparts and reported a greater overall stress (i.e., weekly training strain) during the first week of the training camp. Finally, positive associations were found between the measured training load in week 2 and the probability of being selected to the U18 national team.

Regarding the observed training load variables, no statistically significant between-group differences were found for accumulated training load, total training monotony and strain, and week-to-week changes when comparing early- and average-maturing players. The weekly training load herein (i.e., 7393.31 ± 1301.37 and 6678.96 ± 1416.99 AU for weeks 1 and 2, respectively) was considerably higher than the loads reported in a sample of Italian U17 basketball players (i.e., ~3250 a.u.) [38], possibly due to the differences between conventional in-season training and national team camps, the latter of which is characterised by a short and intense activity schedule. Moreover, when considering playing positions (e.g., most of early-maturing players were inside players, and most of average-maturing players were perimeter players) present findings related with accumulated training load are in line with previous study in elite basketball [39], where sRPE was similar among different playing positions (point guards, forwards, and centres). Furthermore, early-maturing players had a significantly lower training experience and possible influence on perceived training load can be expected [40]. Contrary to findings in Australian football which reported greater sRPE for players with higher playing experience at elite levels (>4 years) compared to less experienced players (<3 years), in the present study accumulated training load was similar between groups [40]. That said, the experience seems to be a mediator of sRPE, especially in contexts where athletes have previous experiences in the same environment, e.g., a professional club [40]. However, in sporadic and a very limited time period settings (e.g., national team training camps), where the experience in this context is similar between subjects, the training experience in the club settings does not seem to influence the accumulated training load. Nevertheless, athletes with less training experience and with lower aerobic fitness (such as early-maturing players) have a greater risk of injury and are even less protected against large variations in training load [18]. This seems to be even more important when the risk of injury is higher in weeks with a high training load (≥2770 AU) [18], as occurs in national team training camp, and early-maturing players (less experienced and with lower aerobic fitness) experienced higher overall stress (i.e., weekly strain), particularly in week 1. Notwithstanding, previous study in Australian Football shown that training strain had a substantial relationship with match outcome [41]. This is particularly interesting when most of early-maturing players experienced higher overall stress during national team training camp and play in inside positions, which is a key position in terms of overall performance (i.e., game efficiency) in FIBA European Youth Championships (Under-16 to Under-20) [42]. That said, practitioners should be aware that experiencing higher overall stress can positively influence the performance of key players and consequently team performance. However, increased weekly strain can be risky in athletes with an ability to tolerate high training loads. Thus, practitioners should develop training load management strategies, for example selecting different types of training, proportion, and duration [38], providing distinct internal load measures in order to provide an “optimal” training load which simultaneously improves players readiness, and decrease in the risk of injury in load sensitive athletes, such as early-maturing players.

When analysing each week separately, the early-maturing group exhibited greater training strain (~33%; ES = 0.74) in week 1 thus indicating that these players reported a higher overall stress during the first week of the camp. The fact that differences were found for training strain, but not training load or monotony when assessed independently, supports previous claims that, in basketball, all three variables should be monitored concurrently on a weekly basis [43]. This is because monotony is more detailed variables which considers the variability in training load across the week. Moreover, these results suggest that early maturing players can experience different response to the applied load during national team training camp. This could be due to coaches placing more pressure on early maturing players during training sessions because they are perceived to be more likely to demonstrate higher performance levels during international competitions. Indeed, more advanced anthropometric characteristics have been shown to be significantly related with game-performance at different levels, particularly in relation to scoring and rebounding parameters. Thus, the tallest, or earlier maturing players, not only may be more likely to be selected, but also to selected in key positions close to the basket in order to increase winning opportunities [11,14]. In this way, during the national training camp, coaches may have adopted a highly motivated demeanour when training more physically advanced players, thus generating increased internal load through additional activity or psychological stress (i.e., sRPE) [44,45].

Maturity timing may be an important aspect to consider to avoid the application of an exacerbated training response during congested training weeks and to reduce injury risk as a high training strain has been associated with illness and overtraining in athletes [32]. This is particularly apt for when early-maturing team-sport players are identified as being at an increased injury risk [46,47] and when musculoskeletal, neuromuscular and physical factors [48,49] might affect load-tolerance [46]. However, to the best of our knowledge, no study has examined training load variables according to maturing timing, thus further studies are recommended to better understanding between those variables, and injury or performance.

Interestingly, according to the present findings, training load in Week 2 was predictive of onward selection to the U18 National Team. The predicted probability of selection was raised alongside greater training load which implies that U16 National Team players that were able to tolerate higher loads were more likely to be selected to the U18 team. A possible explanation for this finding could be seen in the applied training loads which typically increase as athletes progress through the age categories [50]. Because of this trend, players who are exposed to higher workloads at the U16 level may, ultimately, find themselves in a better position to meet and adapt to the demands of training at the more advanced stages of development, thus increasing their probability of being selected to the national team. In this way, it could be that players who learn to sustain higher training loads may become more robust to the future application of intense training stimuli. Such a phenomenon has previously been documented with Hulin and colleagues [51] demonstrating that high and very high workloads ultimately exerted a protective effect against injury in rugby league players. This is an interesting finding in the context of our study as it lends theoretical support to the use of higher workloads in youth athletes as a mechanism for sustained performance and resistance to injury, an admittedly controversial topic in light of the very legitimate concerns with regard to burnout in youth sport [52]. Accordingly, this finding should be treated with great caution until further study can elucidate a clearer relationship between higher workloads and future performance and selection in youth athletes. If this is indeed the case, coaches are still encouraged to use complementary training strategies to improve physical qualities (e.g., strength, speed, aerobic fitness and repeated sprint qualities) that may improve players’ ability to tolerate high workloads and larger week-to-week changes [18,53].

The limitations of the present study should be acknowledged. Firstly, the methods used to calculate MO and PAH are not considered the “gold-standard” and are, therefore, not without inaccuracies [54]. Moreover, previous longitudinal studies analysing MO method, and involving large samples mainly included Caucasian ancestry participants [28], whereas the present study included African ancestry players. Readers should therefore be mindful of this and the potentially discrepancies in MO in these five players. Thereby, the present results should be cautiously interpreted. Nonetheless, these are commonly used methods in research and practice [54]. Secondly, week 2 training load was associated with the probability of an athlete being selected to the U18 National Team but this should be interpreted with caution as several other potentially influential variables were not considered (e.g., other anthropometric variables or physical capabilities). Future research should incorporate external load metrics for better quantification of the players training load as well as develop a more robust predictive model incorporating complementary variables to allow a better understanding of which factors influence future national team selection in young basketball players. Finally, the participants in the current sample are slightly taller and heavier, but also present earlier age of peak height velocity when compared with youth basketball players of an equivalent chronological age in other studies [55,56]. Therefore, the degree to which maturity timing may impact ratings of perceived exertion may vary relative to the nature of the sample.

## 5. Conclusions

This study demonstrates that early-maturing players, who were taller and heavier than their average-maturing counterparts, experienced greater overall stress during the first week of the national basketball team training camp. Consequently, the early-maturing players accumulated a higher training load across the camp, though those of average maturity demonstrated a greater load variation. Practitioners should be mindful of these data when designing, implementing and supervising training sessions and should look to closely monitor TLs during youth national team training camps given that players responded differently to the same load, according to the maturity timing. Regarding the secondary aim of the study, training load in week 2 of the documented camp was associated with onward selection to the U18 national team. Therefore, there appears to be a relationship between the load tolerance of the U-16 national team players during and their future selection to the U-18 team. Practitioners should be mindful that athletes’ ability to cope with high training loads may be crucial to performance. Thus, practitioners are encouraged to develop their athletes’ ability to tolerate high weekly loads.

## Figures and Tables

**Table 1:** Between-group comparisons in the maturity, anthropometrical, and training variables based on the maturity timing (Mean ± SD).

	Early Maturer (*n* = 11)	Average Maturer (*n* = 18)	*p*	Effect Size (ES)
**MATURITY**				
CA (years)	16.04 ± 0.45	16.04 ± 0.28	0.985	
APHV (years)	12.63 ± 0.41	13.64 ± 0.33	**0.000**	−2.80
MO (years)	3.22 ± 0.53	2.33 ± 0.41	**0.000**	1.93
PAH (cm)	196.18 ± 5.24	189.40 ± 5.44	**0.003**	1.26
ANTHROPOMETRICAL				
Height (cm)	195.25 ± 5.52	187.10 ± 6.12	**0.001**	1.38
Sitting height (cm)	98.84 ± 2.81	93.59 ± 1.96	**0.000**	2.27
Body mass (kg)	90.79 ± 9.03	76.09 ± 7.52	**0.000**	1.81
PLAYING POSITIONS				
Perimeter players	23.3%	66.7%		
Inside players	72.7%	33.3%		
YEARS				
2016	63.6%	27.8%		
2017	18.2%	38.9%		
2018	18.2%	33.3%		
U18 NATIONAL TEAM SELECTION				
Yes	54.5%	77.8%		
No	45.5%	22.2%		
TRAINING				
Training experience (years)	5.55 ± 2.38	8.22 ± 2.10	**0.004**	−1.21
Week 1—Training load (a.u.)	7139.82 ± 1248.98	6959.68 ± 1274.32	0.713	
Week 1—Monotony (a.u.)	1.59 ± 0.36	1.24 ± 0.51	0.063	
Week 1—Strain (a.u.)	11,623.64 ± 4014.43	8422.05 ± 3552.59	**0.033**	0.86
Week 2—Training load (a.u.)	8250.64 ± 980.49	8184.06 ± 1632.49	0.904	
Week 2—Monotony (a.u.)	1.51 ± 0.30	1.75 ± 0.35	0.073	
Week 2—Strain (a.u.)	12,528.11 ± 2922.52	14,484.19 ± 4831.89	0.237	
Accumulated Training Load (a.u.)	15,390.46 ± 1885.67	15,097.07 ± 2899.33	0.768	
Total Monotony (a.u.)	1.55 ± 0.17	1.50 ± 0.17	0.404	
Total Strain (a.u.)	23,950.47 ± 4301.00	22,559.76 ± 4663.57	0.430	
Week-to-week absolute difference in load (a.u.)	1110.82 ± 1219.37	1177.71 ± 967.67	0.871	
Week-to-week workload ratio (a.u.)	1.18 ± 0.23	1.18 ± 0.15	0.934	

Abbreviations: CA = Chronological age; APHV = Age at Peak of Height Velocity; cm = centimeters; kg = kilograms; a.u. = arbitrary units; MO = Maturity Offset; PAH = Predict Adult Height. Significant differences at *p* < 0.05.

**Table 2:** Summary of standardized canonical discriminant function coefficients, eigenvalues, and correct classification cases for early and average maturing players in under-16 Portuguese National Team.

Variable	Standardized Canonical Discriminant Function Coefficients
Height	0.437
Sitting Height	0.719
Body Mass	0.574
Training experience	−0.155
Week 1—Strain (a.u.)	0.090
Eigenvalue	2.516
Cases correctly classified	96.6%
Function	^ = 0.284 χ^2^ (3) = 32.065 (*p* = 0.000)

**Table 3:** Summary of stepwise discriminant analyses by selection group: variables entered/remove.

		Entered	Wilks’ Lambda				Exact *F*			*p*
Group	Step	Entered	Statistic	df 1	df 2	df 3	Statistic	df 1	df 2	
Under-18 National Team	1	Week 2—Training load (a.u.)	0.818	1	1	27.0	6.022	1	27.0	0.021

Notes: At each step, the variables that minimizes the overall Wilks’ lambda is entered. Maximum number of steps is 62. Minimum partial *F* to enter is 3.84; maximum partial *F* to remove is 2.71. Legend: df = degrees of freedom.

## Data Availability

The data that support the findings of this study are available from the corresponding author, J.A., upon reasonable request.
